# The Contributions of Domain-General and Numerical Factors to Third-Grade Arithmetic Skills and Mathematical Learning Disability

**DOI:** 10.1037/a0034097

**Published:** 2013-08-19

**Authors:** Richard Cowan, Daisy Powell

**Affiliations:** 1 Psychology and Human Development, Institute of Education University of London, London, England; 2 Institute of Education University of Reading, Reading, England

**Keywords:** mathematics, cognitive correlates, estimation, mathematical learning difficulty, dyscalculia

## Abstract

Explanations of the marked individual differences in elementary school mathematical achievement and mathematical learning disability (MLD or dyscalculia) have involved domain-general factors (working memory, reasoning, processing speed, and oral language) and numerical factors that include single-digit processing efficiency and multidigit skills such as number system knowledge and estimation. This study of 3rd graders (*N* = 258) finds both domain-general and numerical factors contribute independently to explaining variation in 3 significant arithmetic skills: basic calculation fluency, written multidigit computation, and arithmetic word problems. Estimation accuracy and number system knowledge show the strongest associations with every skill, and their contributions are independent of both each other and other factors. Different domain-general factors independently account for variation in each skill. Numeral comparison, a single digit processing skill, uniquely accounts for variation in basic calculation. Subsamples of children with MLD (at or below 10th percentile, *n* = 29) are compared with low achievement (LA, 11th to 25th percentiles, *n* = 42) and typical achievement (above 25th percentile, *n* = 187). Examination of these and subsets with persistent difficulties supports a multiple deficits view of number difficulties: Most children with number difficulties exhibit deficits in both domain-general and numerical factors. The only factor deficit common to all persistent MLD children is in multidigit skills. These findings indicate that many factors matter but multidigit skills matter most in 3rd grade mathematical achievement.

Educational psychologists have long sought to understand the variation in arithmetical skills in the normal range (Spearman, 1927). A more recent interest is in whether the same factors also account for number difficulties (Geary & Brown, 1991). One theoretical perspective explains this variation in terms of cognitive factors, such as working memory, reasoning, processing speed, and oral language (e.g., Fuchs et al., 2006). These are termed domain-general because they influence learning in all domains of knowledge such as mathematics, literature, history, and art.

Another perspective seeks to explain variation in arithmetic by identifying numerical factors that underpin the development and execution of many arithmetical skills. Single digit processing, number system knowledge, and number line estimation are candidate numerical factors. Single digit processing is assessed by magnitude comparison and quantity enumeration in tasks involving up to 10 items. The efficiency of single digit processing, a combination of speed and accuracy, develops with age and is associated with variation in the normal range and number difficulties (Butterworth, 2003, 2010; Reigosa-Crespo et al., 2012). Single digit processing efficiency is proposed to develop relatively independently of domain-general factors. This independence is proposed to explain how number difficulties can be found in children whose domain-general functioning is unimpaired (Landerl, Bevan, & Butterworth, 2004).

Number system knowledge concerns how numbers are related, and number line estimation is the ability to approximate numerical magnitudes (Siegler, 2009). Both show strong relationships with more general measures of arithmetic (Booth & Siegler, 2006; Case et al., 1996; Griffin, 1997, 2005; Siegler & Opfer, 2003). Both are impaired in children with number difficulties (Geary, Hoard, Nugent, & Byrd-Craven, 2008; Gersten, Jordan, & Flojo, 2005; Jordan, Kaplan, Locuniak, & Ramineni, 2007).

Numerical factors such as number system knowledge and number line estimation develop with experience and instruction. As domain-general factors influence learning from experience and instruction, it follows that individual differences in the acquisition of numerical factors will to some extent reflect individual differences in domain-general factors (Case et al., 1996).

The purposes of the present study are to assess the contributions made by domain-general and numerical factors to three significant arithmetic skills in third grade (basic calculation fluency, written arithmetic, and arithmetic word problems) and to examine their role in mathematical learning disability (MLD) and lesser number difficulty.

## Domain-General Factors

Conceptions of general factors have developed from psychometric research and theory. Omnibus intelligence tests (e.g., Wechsler, 1955) include subtests assessing working memory, reasoning, processing speed, and oral language. In the following sections, we consider the basis for expecting each factor to contribute to number development and the issues raised by previous research.

### Working Memory

Working memory is the ability to maintain and process goal-relevant information. Baddeley and Hitch’s (1974) model proposes a limited capacity attentional controller, the central executive, that works in conjunction with two subsystems, one concerned with short-term storage of acoustic and verbal information, the phonological loop, and the other with short-term storage of visual and spatial information, the visuo-spatial sketchpad. It has inspired much research on the correlates of mathematical achievement, but some issues prevent a simple interpretation of existing research as establishing the importance of working memory functioning (Geary et al., 2008; Raghubar, Barnes, & Hecht, 2010). First, there is substantial covariance between working memory and other domain-general factors such as reasoning and processing speed (Burgess, Gray, Conway, & Braver, 2011; Engel de Abreu, Conway, & Gathercole, 2010; Kail & Hall, 2001). This makes it hazardous to interpret associations between working memory measures and mathematical achievement as simply reflecting the importance of working memory functioning.

Second, using numerical activities in working memory tasks, such as counting span (Pickering & Gathercole, 2001) may confound numerical and domain-general contributions: Some studies find children with specific arithmetical difficulties have impaired counting spans but typical listening and comparison spans (Hitch & McAuley, 1991; Siegel & Ryan, 1989). Thirdly, previous research findings are not consistent: This may result from the variation in the measures of working memory and mathematics, the ages of the children, the criteria for identifying low achievement, the language of the children, and the way they are taught (Raghubar et al., 2010).

Despite these issues and concerns about the underlying construct (Towse & Cowan, 2005), most reviews conclude that working memory is relevant to mathematical development and mathematical difficulties (Berch, 2008; Geary, 2011; Raghubar et al., 2010; Swanson & Jerman, 2006; but see Landerl et al., 2004, for a contrasting view).

### Reasoning

Relationships between mathematics and reasoning skill have long been proposed, and mathematical reasoning accounts for differences in mathematics achievement independently of computational skill (Nunes et al., 2007). Raven’s Colored Progressive Matrices (CPM, Raven, 2008) is a domain-general reasoning test that was originally developed to measure a component of *g*, the general factor common to different mental tests (Spearman, 1927). The relation between CPM and mathematical achievement was shown in an epidemiological study of 9- and 10-year-olds (Lewis, Hitch, & Walker, 1994): Most (66%) children with poor achievement in arithmetic had CPM scores in the lowest quartile.

### Processing Speed

Processing speed, the rapidity of execution of mental operations, has been proposed as a general factor underlying individual differences in cognition since Galton. Increases in processing speed have been suggested to underlie age-related cognitive development, including working memory functioning (Fry & Hale, 1996; Kail, 1991). Processing speed accounts for variation in mathematical skills independently of working memory in some studies (e.g., Andersson, 2010; Bull & Johnston, 1997; Chan & Ho, 2010; Fuchs et al., 2006; Hecht, Torgesen, Wagner, & Rashotte, 2001; but note Geary et al., 2008). Processing speed is more related to basic calculation than written arithmetic or story problems (Chan & Ho, 2010; Fuchs et al., 2006).

### Oral Language

Children’s first encounter with numbers is through learning to count and mastering the number word sequence. Oral language is the principal medium of instruction in elementary school. Both suggest that oral language ability is likely to affect the development of mathematical skills and knowledge. Consistent with this, oral language skills independently account for variation in mathematical skills (Cowan, Donlan, Newton, & Lloyd, 2005; Fuchs et al., 2006).

### Summary

All domain-general factors are associated with variation in mathematical skills. Although much variance is shared by domain-general factors, some studies indicate particular factors make unique contributions (e.g., Bull & Johnston, 1997). The contributions depend on the arithmetical skill (e.g., Fuchs et al., 2006). Working memory functioning is often highlighted in relation to arithmetical skills, but this might be because it has been studied more than other domain-general factors.

Relationships between domain-general factors and arithmetical skills might be direct or indirect. Indirect relationships would reflect associations with either numerical factors or other domain-general factors. These possibilities are not exclusive: a zero-order correlation between a domain-general factor and an arithmetical skill might reflect a combination of direct and indirect relationships. By including measures of each domain-general factor and numerical factors in models of variation, this study attempts to assess the nature of the relationships.

## Numerical Factors

The numerical factors we include are single-digit processing, multidigit number system knowledge, and estimation.

### Single Digit Processing

Two accounts of number difficulties assert the importance of single digit processing: the defective number module hypothesis (Butterworth, 2010; Reigosa-Crespo et al., 2012) and the access deficit hypothesis (Rousselle & Noël, 2007). The defective number module hypothesis claims that impairments in single digit processing result from a failure to develop a numerosity system that is initially nonsymbolic, shared with other species, and present in some form at birth. The access deficit hypothesis asserts that the difficulties lie in accessing the meaning of symbols rather than in the nonsymbolic system. Both hypotheses expect speed of numeral comparison to be related to number skills, and this has been found in several studies (Cirino, 2011; Durand, Hulme, Larkin, & Snowling, 2005; Holloway & Ansari, 2009; Reigosa-Crespo et al., 2012). Numeral comparison speed in first grade has also been found to predict mathematics achievement in second grade (De Smedt, Verschaffel, & Ghesquière, 2009).

Studies have mainly supported the access deficit hypothesis. Children with number difficulties are slower in comparing numerals than typically developing children (Andersson & Östergren, 2012; De Smedt & Gilmore, 2011; Iuculano, Tang, Hall, & Butterworth, 2008; Landerl et al., 2004; Rousselle & Noël, 2007), but differences in speed on nonsymbolic tasks have only been reported in one study (Landerl, Fussenegger, Moll, & Willburger, 2009). Some measures of numeral comparison speed use Stroop tasks where the physical size of the numerals is incongruent with their numerical magnitude (e.g., Iuculano et al., 2008). The requirement to inhibit a judgment based on physical size may make the task more sensitive to domain-general factors as inhibition is an aspect of executive functioning (Bull & Scerif, 2001).

Speed of quantity enumeration on sets of up to 10 items is also supposed to reflect the integrity of the number module (Butterworth, 2010). One large sample study finds speed of enumeration to be related to arithmetic (Reigosa-Crespo et al., 2012). Smaller scale comparisons of children with number difficulties do not find consistent differences (Andersson & Östergren, 2012; Landerl et al., 2004). One explanation for the discrepancy may lie in the measures. Reigosa-Crespo et al. (2012) combined both speed and accuracy in their measure, whereas the other studies analyzed speed and accuracy separately.

### Multidigit Skills: Number System Knowledge

The meaning of numbers is determined by their relations to other numbers in the number system. Children typically start school with some knowledge of the number word sequence and the names of numerals (Siegler & Robinson, 1982). During elementary school, they master the system for combining number words and the Hindu-Arabic system for representing numbers with numerals. This enables them to generate accurate counting and numeral sequences from numbers they have not experienced (Skwarchuk & Anglin, 2002).

Number system knowledge has been assessed by the Number Knowledge test (Griffin, 1997, 2005) and count sequence tasks requiring children to count up and down from specified points (e.g., Cowan et al., 2005). Deficient number system knowledge in kindergarten is the best predictor of subsequent mathematics difficulty (Gersten et al., 2005) and, in combination with other measures, number system knowledge predicts later growth in mathematics up to third grade (Jordan et al., 2007; Jordan, Kaplan, Ramineni, & Locuniak, 2009).

Ignorance of place value has been found to discriminate children with mathematics difficulties from their peers (Chan & Ho, 2010), and variation in second grade children’s count sequence knowledge, which principally concerns numbers above 100, substantially correlates with single digit calculation (Cowan et al., 2005).

### Multidigit Skills: Estimation

Estimation is involved in a variety of approximation tasks including judging measurements in standard units, generating ball park answers to computations, and assigning numbers to quantities without counting. Number line estimation is an approximate number task in which a line with numerals at the endpoints is presented and children either estimate the position of target numbers or estimate the number corresponding to target marks.

Although the cognitive mechanisms underlying numeral placements are debated (e.g., Barth & Paladino, 2011; Opfer, Siegler, & Young, 2011), there is no dispute that accuracy of number line estimates correlates substantially with other forms of pure numerical estimation (Booth & Siegler, 2006) and general math achievement (Ashcraft & Moore, 2012; Booth & Siegler, 2008; LeFevre et al., 2010; Schneider, Grabner, & Paetsch, 2009; Siegler, Thompson, & Schneider, 2011).

The development of a mental number line that faithfully represents the relations between numbers has also been suggested to underlie the development of number system knowledge (Case et al., 1996), and the need to understand the number system is recognized for successful estimation (Siegler & Opfer, 2003). Therefore, the extent to which number system knowledge and estimation accuracy independently contribute to mathematics achievement is uncertain. Number estimation has been found to be impaired in children with number difficulties (Geary et al., 2008).

### Summary

General arithmetic skills are related to all the numerical factors. Previous research on single digit processing more often finds relations with numeral magnitude comparison than with quantity enumeration. This may reflect how the measures are derived from performance. In the present study, we combine accuracy with speed to yield efficiency measures. To reduce covariation of single digit processing with domain-general factors, we do not include Stroop trials in the numeral comparison task.

Domain-general factors are hypothesized to affect the development of both number system knowledge and estimation. This yields the prediction of relations between individual variation in domain-general functioning and in these multidigit skills. What is uncertain is the extent to which the multidigit skills account for variation in arithmetical skills independently of domain-general factors and each other. As with the domain-general factors, relationships between numerical factors and arithmetical skills might be direct or indirect, and these are not mutually exclusive. Direct relations would be evidenced by unique contributions to variance, independently of the other factors. Indirect associations might be due to relationships with other numerical or domain-general factors. This study uses models of variation that include the other factors to assess these relationships.

## Arithmetical Skills

This study compares the contributions of general and specific factors to explaining variation in three different measures of arithmetic skill: basic calculation fluency, written multidigit computation, and arithmetic word problems. The rationale for the selection of these is as follows. Basic calculation fluency is chosen as it consistently correlates highly with more general measures of math achievement (e.g., Durand et al., 2005), is frequently impaired in children with math difficulty (e.g., Russell & Ginsburg, 1984), and lastly, is a timed measure as children are only credited for correct answers given in less than 3 s. Basic calculation fluency has always been emphasized in elementary education as it is believed to be crucial for competence in both mental and written arithmetic. As a timed measure, it is likely to be associated with differences in processing speed either at the domain-general level or at the domain specific level of simple number processing.

Written arithmetic involves multidigit computation. Developing competence in written arithmetic remains a key aspiration of the early elementary curriculum and written arithmetic items feature in both curriculum tests and standardized measures of mathematics achievement such as the Wechsler Individual Achievement Test–Second UK Edition (WIAT-II UK; Wechsler, 2005). Written arithmetic is also chosen because individuals can show marked discrepancies between their skill in mental and written arithmetic (Dowker, 2005). This may be because written arithmetic is susceptible to procedural bugs and visuo-spatial deficits (Raghubar et al., 2009). Visuo-spatial deficits were predicted on the basis of early research on number difficulties (Geary, 1993), but subsequent research has yielded less support for them than fact retrieval and procedural deficits (Geary, 2010).

Arithmetic word problems require children to understand a set of verbally expressed propositions, identify the relevant computational problem, and execute it. They are included because competence with word problems has long been perceived by math educators as evidence of the ability to apply arithmetic (Verschaffel, Greer, & De Corte, 2000). Also, they have been the focus of study in previous investigations of cognitive correlates (e.g., Fuchs et al., 2006; Swanson & Beebe-Frankenberger, 2004). Word problems accuracy is more related than basic calculation proficiency to oral language skill and general ability, and this is consistent with the difference in cognitive demands made by these skills (Fuchs et al., 2006).

On the basis of previous work, we anticipate that across the three outcomes the domain-general factors will vary in their contributions. In contrast, we hypothesize that both number system knowledge and estimation will contribute to all three arithmetical outcomes. The theory behind number system knowledge (Siegler, 1996) posits a bidirectional relationship between conceptual knowledge about the number system and even simple computational skill. For example, insight into the number sequence yields knowledge of arithmetical facts such as *n* + 1, *n* − 1, and *n* − (*n −* 1). Place value understanding is important for understanding written arithmetic procedures. As estimation skill supports computational estimation then it should support successful monitoring of the execution of procedures in written arithmetic.

## Mathematical Learning Disability (MLD) and Degrees of Number Difficulty

Mathematical learning disability (MLD, Geary, 2011) is acknowledged to be the same construct as mathematics disorder (*Diagnostic and Statistical Manual of Mental Disorders,* 4th ed.; *DSM–IV*; American Psychiatric Association, 1994), developmental dyscalculia (Butterworth, 2010; Wilson & Dehaene, 2007), and specific arithmetic difficulties (Lewis et al., 1994). All terms refer to cases where poor arithmetic performance is combined with at least average intelligence. Identifying qualitative discontinuities or biological markers for MLD would enable researchers to escape the arbitrariness of purely statistical criteria, but these have yet to be discovered.

Comparisons of groups varying in number difficulties have indicated both domain-general and numerical factors are relevant (Geary, 2011). This could be due to multiple deficits being required for number difficulties, as has been argued for reading (Pennington, 2006) and oral language (Bishop, 2006). Alternatively, it might reflect heterogeneity in the deficits that can give rise to number difficulties. While some children’s difficulties may be due to a combination of domain-general and numerical factors, others may result solely from deficits in single-digit processing (Butterworth & Yeo, 2004).

The findings from behavioral-genetic studies have been interpreted as consistent with numerical factors as responsible for number difficulties (Butterworth, Varma, & Laurillard, 2011). Although much genetic variation is shared between disabilities in arithmetic, oral language, and reading, there is some genetic variation specific to arithmetic (Kovas, Haworth, Harlaar, Petrill, Dale, & Plomin, 2007). Unique genetic contributions to arithmetic are also consistent with influences on domain-general factors: Some domain-general factors may play a greater role in arithmetic than in reading or oral language. Visuo-spatial functioning might be such a domain-general factor: Impaired visuo-spatial functioning has been found in children who combine poor math with average reading (McLean & Hitch, 1999; Swanson, 2012) and has been suggested as responsible for a subtype of number difficulties (Geary, 1993; Wilson & Dehaene, 2007; but see Geary, 2010, for a contrasting view).

MLD has been identified on the basis of arithmetic below the 16th percentile (e.g., De Smedt & Gilmore, 2011; Landerl et al., 2009; Oliver et al., 2004) but some consensus is emerging in defining MLD as scores below the 11th percentile, identifying scores between the 11th and 25th percentiles as low average or low achievement (LA) and treating any scores above the 25th percentile as typical achievement (TA, Geary, Hoard, Byrd-Craven, Nugent, & Numtee, 2007; Geary, Hoard, Nugent, & Bailey, 2012; Mazzocco, Feigenson, & Halberda, 2011; Mazzocco & Myers, 2003; Murphy, Mazzocco, Hanich, & Early, 2007).

Previous studies differ in the type of arithmetic test used to identify number difficulties, the IQ cutoff score, and the persistence of number difficulties (Murphy et al., 2007). Whether timed or untimed tests should be used is a matter of debate (Berch, 2005; Gersten et al., 2005). Although some argue that timed tests should be used (e.g., Butterworth, 2003), this can lead to children being identified as dyscalculic on the basis of timed tests whose classroom math and performance on untimed tests are normal (Reigosa-Crespo et al., 2012).

Using an IQ cut off is consistent with the clinical diagnosis of MLD (Geary, 2011), but it excludes the majority of children with poor math (Lewis et al., 1994), and it is likely to attenuate the influence of domain-general factors that correlate with IQ. Also where the cutoff should be made varies with some studies excluding only children with IQs less than 70, consistent with World Health Organization (1993) recommendations, and others using higher cutoffs (Murphy et al., 2007). Geary (2011) recommends a cut off at 85, that is, the 15th percentile.

Persistence can be determined from consistency across two consecutive academic years (Geary, 2011). Requiring persistence should make research samples more similar to clinical samples as persistence is required for diagnosis (World Health Organization, 1993). On the other hand, requiring persistence reduces sample sizes and may accentuate differences between groups.

In this study, we examine the occurrence of domain-general and numerical deficits in MLD, LA, and TA groups. First, we construct the groups on the basis of a standardized untimed test administered in third grade. Secondly, we examine the subset of these children with persistent difficulties and exclude those with IQs below 85.

## Aims of the Study

The main aim of this study is to assess the relationships of domain general and domain specific factors to variation in mathematical achievement. We use measures of general factors that do not involve numbers so as to eliminate the contribution of number knowledge and skills from domain-general assessments. As domain-general functioning affects assessment of numerical factors, fixed order regressions are used to ascertain the contributions of each type of factor independently of the other.

The second aim of this study is to assess the contribution of deficits in domain-general and numerical factors to MLD, LA, and TA group membership and differentiation. We examine groups defined by single point assessment and subsets with persistent difficulties to enhance comparability with other studies and prevent conclusions that are specific to the method of group construction.

## Method

### Participants

The sample comprised 258 (125 male, 123 female) participants in a longitudinal math project. They were drawn from nine state school classes in the same English administrative district. When assessed in second grade, their ages ranged from 7 years 0 months to 9 years 5 months (*M* = 7 years 11 months, *SD* = 5 months), and at the third grade assessments, their ages ranged from 8 years 0 months to 10 years 5 months (*M* = 8 years 11 months, *SD* = 5 months). The large range in age results from assessments taking place throughout the school year and a child who was 8 months older than the others.

The proportion claiming free school meals was 5.6%, which is average for the source administrative district but below the national average (13.1%: Department for Children, Schools and Families, 2007). Children are provided with additional support with classroom learning if their administrative district issues them with statements of special educational needs or if their school identifies them for action because they are making poor academic progress. The sample included nine children with statements and a further 39 children identified for school action.

### MLD, LA, and TA Groups

Groups varying in number difficulties were first constructed from third grade mathematical composite scores of the WIAT-II UK (Wechsler, 2005): MLD for scores below 82, LA for scores between 82 and 90, and TA for scores above 90. The validity of this as an indicator of general mathematical performance is suggested by its substantial correlation (*n* = 257, *r* = .77) with teachers’ assessments of children’s number skills using a national scheme. In these single point assessment groups there were 29 MLD (13 girls, 16 boys), 42 LA (27 girls, 15 boys), and 187 TA (85 girls, 102 boys) children. The numbers of children qualifying for additional support through statements or being identified for school action were as follows: 17 in the MLD group, 9 in the LA group, and 22 in the TA group.

The persistent groups were the subsets of the single point assessment groups that scored in the same percentile range on a basic calculation fluency test administered in second grade and had a standard score of 85 or more on the CPM (Raven, 2008). For example a child was only included in the persistent MLD group if they were in the single point MLD group, their second grade basic calculation fluency score was below the 11th percentile, and their CPM standard score was 85 or more.

The validity of the second grade assessment is indicated by its concurrent correlation with teachers’ assessments of a subset of children in second grade (*n* = 212, *r* = .76), and its predictive relations with the third grade mathematical composite score (*n* = 258, *r* = .78) and third grade teachers’ ratings (*n* = 257, *r* = .71). The additional requirements for persistent group membership resulted in 67 children being excluded: 14 due to low CPM scores, 44 because of inconsistency between second grade and third grade, and 9 due to both low CPM and inconsistency. In the persistent and specific groups there were 11 MLD (7 girls, 4 boys), 14 LA (8 girls, 6 boys), and 166 TA (75 girls, 91 boys) children. The frequencies of children qualifying for additional support were as follows: 6 in the MLD group, 6 in the LA group, and 17 in the TA group.

### Materials and Procedure

#### Domain-general factors

We assessed the three working memory components with subtests of the Working Memory Test Battery for Children (WMTB-C, Pickering & Gathercole, 2001): phonological loop (Word List Recall), visuo-spatial sketchpad (Block Recall and Mazes Memory), and the central executive (Listening Recall). Reasoning was assessed with the CPM (Raven, 2008). Processing speed was measured with the Symbol Matching subtest of the Wechsler Intelligence Scale for Children (Wechsler, 1992) and the Pair Cancellation test from Woodcock-Johnson III (W-J III, Woodcock, McGrew, & Mather, 2001). To assess Rapid Automatized Naming (RAN) we used the Rapid Letter Naming subtest of the Comprehensive Test of Phonological Processing (CTOPP, Wagner, Torgesen, & Rashotte, 1999). Oral language skills were assessed with the electronic version of the Test for Reception of Grammar–Version 2 (TROG-E, Bishop, 2005) and the British Picture Vocabulary Scale (BPVS II, Dunn, Dunn, Whetton, & Burley, 1997). None of the domain-general tests featured numerical stimuli or activities.

#### Numerical factors

##### Single digit processing

###### Quantity enumeration

The computer-presented Dot Enumeration test from the Dyscalculia Screener (Butterworth, 2003) was used. Participants had to decide whether the numerosity of dots on the left half of the screen matched the numeral on the right. The numerosities and numerals varied from 1 to 10. There were 68 test trials. Efficiency scores were obtained by dividing the median time for correct responses by percentage accuracy.

###### Numeral comparison

The measure of numeral comparison speed was derived from the Numerical Stroop test in the Dyscalculia Screener (Butterworth, 2003). Participants had to identify the larger of two numerosities represented by single digit numerals. The full test includes trials where the numerals differ in physical size either consistently or inconsistently with the numerosities represented. We only used data from the 10 trials involving numerals that are the same physical size. Efficiency scores were obtained by dividing the median time for correct responses on these trials by percentage accuracy.

##### Multidigit skills

###### Number system knowledge

The test included several types of item: number naming (e.g., “What number comes *five numbers after 49*?”), relative magnitude (e.g., “Which is more? 69 or 71?”), and numerical distance (e.g., “Which number is closer to *49: 51 or 45?*”). All these were derived from the Number Knowledge Test (Griffin, 1997). We also used oral items that required children to recite number sequences, such as from 194 to 210 and from 325 to 317 and written number sequence items that required children to continue sequences of numerals such as from 899 to 901 and from 70,001 to 69,999. There were 32 items in total.

###### Estimation

We used a number line test derived from Booth and Siegler (2006, Experiment 2). Children estimated the position of 22 numbers on a scale from 0–1,000. A different 25 cm number line was used for each estimate. The target numbers were presented in the same order to all children: 475, 690, 297, 103, 721, 158, 391, 3, 874, 586, 240, 835, 502, 962, 19, 346, 907, 7, 438, 52, 613, and 760. Estimates were converted into numerical values corresponding to their position on the scale. Mean percent absolute errors were calculated as in Booth and Siegler (2006).

#### Arithmetic skills

##### Basic calculation fluency

Children’s basic calculation fluency was assessed with a forced retrieval task that presented them with addition and subtraction combinations involving addition of numbers up to 10 and subtractions from numbers less than 20. Combinations were presented one at a time on a laptop computer. As each item was displayed, it was read out by the experimenter. To be judged correct, the child had to answer correctly within 3 s from when the experimenter finished reading out the problem. There were 28 items.

##### Written arithmetic

Skill in addition and subtraction was assessed with 15 computation problems involving two and three digit numbers. The items increased in difficulty from two digit number problems involving no carrying or borrowing to three digit number problems. They were presented on a single sheet and children were encouraged to attempt each one. The items were as follows: 23 + 44, 68 – 42, 26 + 67, 62 − 14, 45 + 28, 75 – 38, 235 + 142, 247 − 247, 613 + 324, 326 − 125, 523 + 168, 894 – 513, 349 + 234, 681 – 214, and 572 − 348. Testing was discontinued if a child indicated he or she were unwilling to attempt any more problems.

##### Arithmetic word problems

Proficiency with arithmetic word problems was assessed with the Mathematical Reasoning subtest from the Wechsler Individual Achievement Test–Second UK Edition (WIAT-II UK, Wechsler, 2005). The subtest is an orally presented verbal problem-solving test with pictures.

### Procedure

In second grade, we assessed working memory, processing speed, oral language, number system knowledge, single digit processing, and basic calculation fluency. In third grade, we conducted the assessments of reasoning, RAN, estimation, written arithmetic, and arithmetic word problems and reassessed basic calculation fluency.

For each child, the interval between second and third grade assessments was between 11 and 13 months, with 12 months being the commonest interval (73%). One child had an interval of 14 months. Testing was conducted by a female researcher during the school day.

## Results

The aims of the study are addressed in two sets of analyses. The first set assesses the contributions of domain-general and numerical factors to explaining variation in the three arithmetical skills. The second examines which factors discriminate between MLD and LA and between LA and TA.

Table 1 shows the descriptives for each factor measure. Indicators of internal reliability are Cronbach alphas for accuracy measures. For the single digit processing measures, reliability estimates are derived by applying the Spearman-Brown formula to correlations between odd and even trial medians. For subsequent analyses, composites were formed for visuo-spatial sketchpad, processing speed, and oral language by averaging the standardized scores of constituent measures. Forming a visuo-spatial sketchpad composite from the Block Recall and Mazes Memory tests is justified by their combination in the WMTB-C to form a visuo-spatial sketchpad component (Pickering & Gathercole, 2001) and the correlation between scores in the present sample (*r =* .39). The processing speed constituent measures correlated substantially (*r* = .53), as did the oral language constituents (*r =* .54). Single digit efficiency scores and RAN were transformed into speed measures so that higher scores meant faster performance. Measures were transformed to deal with deviations in normality as suggested by Tabachnick and Fidell (2007). They were each regressed onto a composite of chronological age and month of testing (cf. Cahan & Cohen, 1989; Cahan, Greenbaum, Artman, Deluy, & Gappel-Gilon, 2008). The standardized residuals from these regressions were treated as the factor variables.[Table-anchor tbl1]

Table 2 shows the correlations between the factor variables. Consistent with previous research, correlations between domain-general factors mostly indicated moderate effects (.30 < *r* < .50, Cohen, 1988), except for RAN, which only substantially correlated with processing speed. Both multidigit skills correlated substantially with general factors, apart from RAN and phonological loop, and were strongly correlated (*r* > .50) with every arithmetic skill and with each other. By contrast single digit processing skills were less related to general factors and arithmetic skills with quantity enumeration showing weaker relationships than numeral comparison.[Table-anchor tbl2]

As children were drawn from different school classes we ran one-way analyses of variance with school class as a fixed factor for each factor variable to determine the extent of clustering. The intraclass correlations (ICC) were calculated from these in accordance with Cohen, Cohen, West, and Aiken (2003). They are reported in Table 1. Most factor variables show no evidence of clustering, but there is some risk of alpha inflation from the factors that did vary with school class. School class is not a source of variation of interest in this study, so we eliminate it by regressing each factor variable against school class coded as a set of dummy variables. The ICCs for the school class residualized versions of factor variables are all zero. Although the correlations between factor variables and their school class residualized versions are very high, all greater than .93, we repeat the analyses using the school class residualized versions to check whether they identify the same factors.

### The Contributions of Domain-General and Numerical Factors to Arithmetic Skills

We conducted sets of fixed-order regression analyses to assess the contributions of domain-general factors, multidigit number skills, and single digit processing. All factors were included in these analyses because comparing their contributions both within and across arithmetical skills is the purpose of this study rather than identifying the most parsimonious set of variables.

#### Basic calculation fluency

Table 3 summarizes the results of the fixed-order regression analyses. It shows that domain-general factors accounted for variance whether they were the first or last block to be entered, but the amount of variance varied considerably with order of entry (first 36%, last 3%). Much of the variance accounted for by domain-general factors was shared between them. When domain-general factors were the first block to be entered (Models A-1 and B-1), the factors that uniquely accounted for variance were processing speed (6%, *t =* 5.01, *p* < .001), oral language (1%, *t =* 2.04, *p =* .043), and RAN (2%, *t =* 3.11, *p* = .002). When domain-general factors were entered last, only processing speed still uniquely accounted for variance (1%, *t =* 2.48, *p* = .014).[Table-anchor tbl3]

Multidigit skills accounted for much of the variance in basic calculation fluency (54%) when they were entered first (Model D-1), and although there was much shared variance, both factors made unique contributions; number system knowledge (21%, *t* = 10.89, *p* < .001) and estimation (2%, *t* = 3.03, *p =* .003). Both still made unique contributions when entered last (Model A-3); number system knowledge (11%, *t* = 7.94, *p* < .001) and estimation (1%, *t =* 2.50, *p =* .013).

The single digit processing factors differed: numeral comparison was a significant unique predictor whatever other factors were in the model but quantity enumeration never was: Model C-1, numeral comparison (14%, *t* = 6.82, *p* < .001) and quantity enumeration (*t =* 1.84, *p =* .067); Model A-2, numeral comparison (3%, *t* = 4.34, *p* < .001) and quantity enumeration (*t =* 0.02, *p =* .981); Model D-2, numeral comparison (2%, *t* = 3.53, *p* < .001) and quantity enumeration (*t* = −0.05, *p =* .958): Model B-3, numeral comparison (1%, *t* = 2.48, *p =* .014) and quantity enumeration (*t* = −0.65, *p =* .519).

In summary these analyses indicate that both number system knowledge and estimation are substantial predictors of basic calculation fluency. Their contribution is independent of variance shared with single digit processing and general factors. Processing speed and numeral comparison are the only other factors to contribute independently of other factors. The same results were obtained when the school class residualized versions of factor variables were used.

#### Written arithmetic

The contributions of domain-general and domain-specific factors are summarized in Table 4. Domain-general factors account for more variation in written arithmetic (43%) than in basic calculation fluency (36%). The subset of domain-general factors uniquely accounting for variance in written arithmetic differ from that for basic calculation fluency: When entered first (Models A-1 and B-1), the unique predictors are visuo-spatial sketchpad (2%, *t =* 2.97, *p* = .003), central executive (1%, *t =* 2.11, *p* = .036), reasoning (4%, *t =* 4.08, *p* < .001), RAN (1%, *t =* 2.05, *p* = .041), and oral language (2%, *t =* 2.88, *p =* .004). As with basic calculation fluency the shared variance is considerable. When entered last, the only significant predictors are visuo-spatial sketchpad (*t =* 2.44, *p* = .015), reasoning (*t =* 2.59, *p* = .010), and oral language (*t =* 2.10, *p =* .037), and they account for 1% of variance each.[Table-anchor tbl4]

Both multidigit skills factors uniquely contribute to variance in written arithmetic whether entered first or last: Model D-1, number system knowledge (11%, *t* = 7.13, *p* < .001) and estimation, (5%, *t* = 4.66, *p* < .001); Model A-3, number system knowledge (4%, *t* = 4.54, *p* < .001) and estimation (1%, *t =* 2.54, *p =* .012). Again much of the variance is shared between predictors.

Quantity enumeration never uniquely accounts for variance. Numeral comparison only makes a unique contribution to written arithmetic when multidigit skills are not included: Model C-1, numeral comparison (6%, *t* = 4.14, *p* < .001) and quantity enumeration (*t =* 1.34, *p =* .182); Model A-2, numeral comparison (1%, *t* = 2.60, *p =* .010) and quantity enumeration (*t =* −0.82, *p =* .414); Model D-2, numeral comparison (*t* = 1.01, *p =* .316) and quantity enumeration (*t* = −0.75, *p =* .457): Model B-3, numeral comparison (*t* = 1.07, *p =* .285) and quantity enumeration (*t* = −1.41, *p =* .160).

In summary, the written arithmetic analyses concur with the basic calculation fluency analyses in identifying both number system knowledge and estimation as making important contributions that are independent of other factors. Unlike basic calculation fluency, the domain-general factors identified as significant are the visuo-spatial sketchpad component of working memory, reasoning, and oral language. Neither single digit processing factor contributes independently of other factors. Broadly, the same results were obtained when using the school class residualized versions. The only differences were that reasoning did not make a significant contribution in models that included the multidigit skills.

#### Arithmetic word problems

The results of the fixed-order regressions are summarized in Table 5. Domain-general factors account for even more variance in arithmetic word problems than in written arithmetic when entered first and the factors making unique contributions are different: When entered first (Models A-1 and B-1), the unique predictors are central executive (2%, *t =* 3.49, *p* = .001), reasoning (3%, *t =* 4.23, *p* < .001), processing speed (3%, *t =* 3.93, *p* < .001), and oral language (3%, *t =* 4.21, *p* < .001), but the amounts of variance they account for are small (2%–4%). When entered last, the only significant predictors are central executive (1%, *t =* 2.50, *p* = .013), reasoning (1%, *t =* 2.09, *p* = .037), and oral language (2%, *t =* 3.61, *p* < .001).[Table-anchor tbl5]

Both number system knowledge and estimation uniquely account for variance in arithmetic word problems whether entered first or last: Model D-1, number system knowledge (12%, *t* = 8.57, *p* < .001) and estimation (9%, *t* = 7.39, *p* < .001); Model A-3, number system knowledge (3%, *t* = 4.78, *p* < .001) and estimation (4%, *t =* 5.61, *p* < .001).

Similar to written arithmetic, neither single digit processing skill makes a unique contribution when multidigit skills are included. Unlike the other arithmetic skills, quantity enumeration does make a contribution independently of numeral comparison but only when no other factors are included: Model C-1, numeral comparison (9%, *t* = 5.72, *p* < .001) and quantity enumeration (1%, *t =* 2.11, *p =* .036); Model A-2, numeral comparison (2%, *t* = 3.39, *p =* .001) and quantity enumeration (*t =* −0.09, *p =* .932); Model D-2, numeral comparison (*t* = 1.50, *p =* .135) and quantity enumeration (*t* = −0.33, *p =* .745): Model B-3, numeral comparison (*t* = 1.54, *p =* .125) and quantity enumeration speed (*t* = −1.02, *p =* .307).

Overall the analyses indicate that both number system knowledge and estimation are important for arithmetic word problems and each explains more variance than any other factor. The contribution of domain–general factors is substantial and is only partially mediated by numerical factors. Neither single digit processing factor makes a unique contribution when multidigit skills are included in the model. The same results were obtained when the school class residualized versions of factor variables were used.

### Discriminating MLD From LA and LA From TA

Previous research finds both domain-general and numerical factors differentiate MLD from LA and LA from TA. Here we analyze the role of domain-general and numerical factors in number difficulties by comparing group means and the incidence of domain-general and numerical deficits in the single point and persistent groups. The group means for IQ (CPM standard scores) and mathematics achievement (WIAT-II UK Mathematics Composite) are shown in Table 6.[Table-anchor tbl6]

All factors showed overall group differences: single point, *F*(2, 255) ≥ 11.37, *p* < .001; persistent, *F*(2, 188) ≥ 6.13, *p* < .005. Table 6 shows all three groups differed in number system knowledge and estimation accuracy in both the single point groups and persistent subsets. Also consistent across both classifications were the differences between the MLD and LA groups in numeral comparison efficiency and the differences between LA and TA groups on reasoning, processing speed, and central executive functioning.

**Figure 1 fig1:**
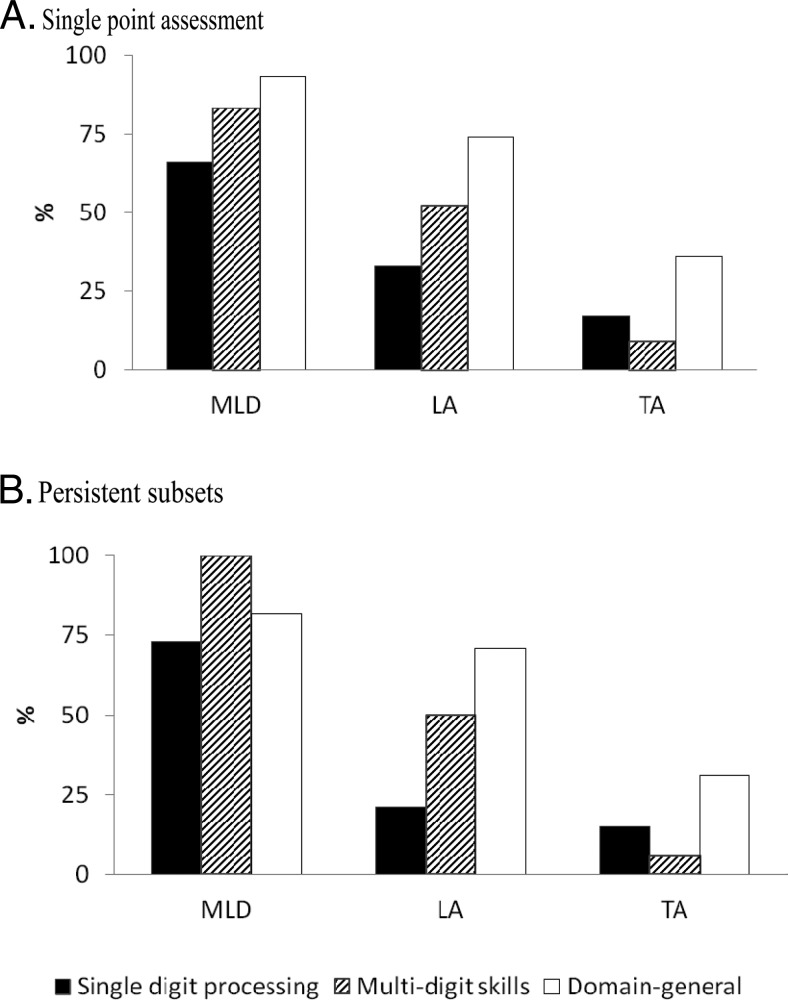
Percentages of mathematical learning disability (MLD), low achieving (LA), and typical achieving (TA) groups with domain-general and numerical factor deficits: single point assessment (A) and persistent subsets (B).

Another way of considering the contribution of domain-general and numerical factors is through examining the incidence of deficits in the groups. We categorized children as having deficits if their score on one or more of the constituent factors was more than 1SD below the mean. Figure 1 shows the proportions of deficits in single digit processing, multidigit skills, and domain-general factors in each group. Domain-general deficits were common even in the persistent subsets that excluded children with low CPM scores. Multidigit skills deficits vary most with group in both methods of constructing groups.[Fig-anchor fig1]

In both single point and persistent classifications, the MLD groups had higher proportions of single digit processing and multidigit skills deficits than the LA groups, but the frequency of domain-general deficits did not differ: single digit processing, single point χ^2^(1, 71) = 5.91, *p* = .015, persistent χ^2^(1, 25) = 4.66, *p* = .031; multidigit skills, single point χ^2^(1, 71) = 5.67, *p* = .017, persistent χ^2^(1, 25) = 5.36, *p* = .021; domain-general, single point χ^2^(1, 71) = 3.08, *p* = .079, persistent χ^2^(1, 25) = 0.02, *p* = .895.

The LA groups had more multidigit skills deficits and domain-general deficits than the TA groups in both classifications, but single digit processing deficits were more common only in the single point groups: single digit processing, single point χ^2^(1, 229) = 5.08, *p* = .024, persistent χ^2^(1, 180) = 0.10, *p* = .755; multidigit skills, single point χ^2^(1, 229) = 42.48, *p* < .001, persistent χ^2^(1, 180) = 24.279, *p* < .001; domain-general, single point χ^2^(1, 229) = 18.69, *p* < .001, persistent χ^2^(1, 180) = 7.82, *p* = .005.

Most children in the MLD and LA groups had more than one type of deficit. In the single point groups, 86% (25/29) MLD and 55% (23/42) LA children had multiple deficits. This pattern was repeated in the persistent subset: 91% (10/11) of the MLD group and 50% (7/14) of the LA group had combinations of deficits. No MLD children just had single digit processing deficits. There was one MLD child who only had deficits in multidigit skills, and she was in the persistent subset. In the LA groups there were two cases of single digit processing deficits in isolation, but neither were in the persistent subset. There were three children in the single point LA group whose only deficits were in multidigit skills, and one of these was also in the persistent subset.

In summary, multidigit skills differentiated most clearly between number difficulty groups. In our sample, the overwhelming majority of low achievement and mathematical learning disability groups had both domain-general and numerical factor deficits. No children in the persistent subsets of MLD and LA groups had single digit processing deficits in isolation.

## Discussion

This study contributes to knowledge about arithmetical skills and children with low mathematical achievement in several ways. First, it establishes that domain-general factors are directly important in determining variation in arithmetical skills. This broadly endorses the conclusions drawn by others (e.g., Fuchs et al., 2006; Geary, 2011; Gersten et al., 2005) but adds to previous work by showing that their contribution is not mediated wholly by numerical factors and that working memory is not the only general factor to be important. Secondly, it indicates arithmetical skills vary in their relations to domain-general factors. This extends the findings of Fuchs et al. (2006) as we assessed the contributions of domain-general factors independently of their relations to the numerical factors in our model. Thirdly, it establishes that although the multidigit skills of number system knowledge and estimation are substantially related, both are important in their own right in explaining variation in every arithmetical skill. Fourthly, almost all children in the number difficulty groups exhibit deficits in both domain-general factors and numerical factors, in particular multidigit skills. This obtains even when a domain-general factor is used to exclude children. Finally numeral comparison seems more relevant to variation in arithmetical skills and number difficulties than the other single digit processing skill, quantity enumeration. This seems more consistent with the access deficit than the defective number module account of the importance of single digit processing.

Before we discuss the interpretations of the results further, we must acknowledge the limitations of our study. The foremost of these is that we have studied a single cohort of children drawn from one grade for 1 year. It is quite possible that the findings might vary with year of schooling. Another limitation is that the sample sizes for our persistent number difficulty groups are small. This reduces the power to detect relationships. Another constraint is that the conclusions about the importance of factors do depend on the quality of the measures used to assess them. Finally although the factors we include do broadly represent the factors identified as salient in previous research, it is possible that further research will identify numerical factors that explain the variance we attribute to domain-general factors or domain-general factors that account for the variance attributed to numerical factors.

### Domain-General Factors

The use of domain-general measures that do not feature numerical stimuli or activities and the inclusion of multidigit skills and single digit processing in models strengthen the support that this study offers for the role of domain-general factors in arithmetic skills and in discriminating between groups. Attributing importance to one domain-general factor rather than another is complicated by the ways in which tasks ostensibly assessing one factor draw on skills implicated in others.

Central executive measures such as listening recall and backward digit span consist of a phonological loop task and extra processing (Savage, Lavers, & Pillay, 2007). This reduces the likelihood of finding direct relationships between phonological loop functioning and outcomes in models that include central executive measures. There are also connections between working memory tasks and other general factors. Phonological loop and central executive measures draw on oral language skills through using verbal material. Faster processing speed supports faster responding and hence reduces the storage time required on all working memory tasks.

Conversely, measures of other general factors make demands on working memory. The processing speed tasks we used require children to remember symbol combinations while searching for duplicates. The reasoning and oral language tasks require children to identify the best solution from a set of choices. These give grounds for expecting correlations between measures of domain-general factors. They also suggest caution in interpreting findings from studies like ours that feature several general factor measures in addition to working memory as contradicting those that have concentrated on working memory.

These considerations suggest that it may be difficult to disentangle domain-general factors in correlational studies. The zero-order correlations show all domain–general factors are related to each other as well as to all the arithmetical skills. They are also related to every numerical factor: this challenges the notion that numerical factors are independent of domain-general factors. Unique contributions in the regressions suggest direct relationships. The factors identified as making direct relationships varied with arithmetical skill in broadly explicable ways. Basic calculation fluency does depend on speed of response, so a direct relationship with processing speed is plausible. Similarly the importance of visuo-spatial sketchpad for written arithmetic involving multidigit numbers is consistent with the role it is expected to play in keeping place when reading (Baddeley, 2003). The importance of oral language, reasoning, and central executive for arithmetic word problems may reflect the demands these make: solving arithmetic word problems requires understanding the language in which the problem is expressed, identifying the operations required, and remembering the question while working out the answer.

Domain-general factors are more important for variation in written arithmetic and word problems than basic calculation fluency. This suggests that solving written arithmetic and word problems is more than just the execution of routine arithmetic skills and procedures. This might well change with school year as a result of practice. With increasing practice, domain-general factors may make less of a contribution to written arithmetic. Future research will determine whether this decline occurs and whether it applies to word problems too.

The importance of domain-general functioning was also indicated by the analyses of number difficulty groups. Domain-general deficits in isolation did not prevent typical achievement, as Figure 1 shows, but most children in MLD and LA groups showed combinations of general and numerical factor deficits. This was even true in the persistent subsets that excluded children with low IQs. The findings support a multiple deficit characterization of number difficulties.

### Multidigit Skills

Understanding the natural number system as expressed in number words and numerals is hypothesized to be a crucial component of number development (e.g., von Aster & Shalev, 2007). Case et al. (1996) proposed that an internal representation of the system constitutes the core conceptual structure for number, and measures of number system knowledge are included in the number sense battery developed by Jordan et al. (2007). Previous studies have shown number system knowledge in kindergarten is an important predictor of number skills a year later. Our study extends these results to older children and establishes that number system knowledge is important for each of the arithmetical skills independently of its relations to domain-general factors.

Number system knowledge and estimation accounted for variance independently of each other despite both being regarded as assessing the quality of an internal mental number line. There are several possible explanations for this to be explored in further research. One possibility derives from considering the arithmetical competences underlying successful performance on the measures. The number system knowledge test involves no more than addition and subtraction. To make accurate estimates may require understanding ratios and division.

Another possibility is that the difference reflects the approximate nature of estimation. Some of the variation in estimation skill may reflect differences in acuity of the approximate number system (ANS) that has been found to discriminate MLD from LA groups (Mazzocco et al., 2011). Although the ANS is held to be present at birth in nonsymbolic form and be common to other species, it undergoes substantial refinement during development (Halberda & Feigenson, 2008). Relations between ANS functioning and number line estimation are likely but have yet to be studied.

### Single Digit Processing

Numeral comparison and quantity enumeration are suggested to measure a system that is a cognitive precursor for the development of more advanced number skills (Butterworth, 2010; Reigosa-Crespo et al., 2012). It is difficult to reconcile this view with our finding that arithmetical skills are more strongly related to numeral comparison than to quantity enumeration and that only numeral comparison differentiated persistent MLD from LA. These findings could be reconciled with the access deficit hypothesis (Rousselle & Noël, 2007) if the quantity enumeration task does not require the relevant understanding of number: recognizing that the numeral 3 matches the number word resulting from quantifying a set of three objects may be achieved without accessing the quantitative meaning of numerals or number words. Certainly young children can count sets without understanding number (e.g., Piaget, 1952). They can name numerals without doing so either. Automatically accessing the quantitative meaning of numerals may not occur until fifth grade (Girelli, Lucangeli, & Butterworth, 2000; but see Berch, Foley, Hill, & Ryan, 1999, and Rubinsten, Henik, Berger, & Shahar-Shalev, 2002, for contrasting views).

In our study, numeral comparison is only directly related to one arithmetical skill, basic calculation fluency. Direct relations with every skill would be expected if numeral comparison efficiency reflected a characteristic of general importance for number development. The small and specific contribution of numeral comparison in our study might reflect the age of our sample: single digit processing in first grade predicts second grade math (De Smedt et al., 2009), but single digit processing is unrelated to mathematical achievement in fifth and sixth grade (Schneider et al., 2009).

The relation between numeral comparison efficiency and basic calculation fluency might result from both being affected by calculation experience. Calculation experience is a major driver of the development of calculation fluency (Shrager & Siegler, 1998). Calculation experience can involve comparing numbers, as in determining the smaller of two addends so that a more efficient counting strategy may be used. Another possibility is that numeral comparison efficiency might affect strategy development: the ease with which the smaller of two addends is identified may affect the shift toward more economical strategies.

Single digit processing deficits were more common in MLD than LA but did not discriminate persistent LA from TA. No child in the MLD groups or persistent LA subset had an isolated deficit in single digit processing. These groups have small sample sizes, and so this finding must be interpreted with caution, but it is the only study so far to include a range of domain-general factors as well as single digit processing measures. It does not support the idea that most children with MLD have isolated deficits in single digit processing and shows that IQ cutoffs do not rule out general factor deficits.

### Conclusions and Implications

This study finds that both domain-general and numerical factors make important contributions to arithmetic skills and to number difficulties. Although this study used measures in 1 year to predict differences a year later, it does not escape the limitations of a correlational design. The case for the causal significance of factors can be strengthened by the success of interventions that focus on them in producing general gains in mathematical achievement.

All domain-general factors are modifiable, but the evidence of the benefits for mathematical development is limited and controversial (Holmes, Gathercole, & Dunning, 2009; Mackey, Hill, Stone, & Bunge, 2011; Shipstead, Redick, & Engle, 2010). Both number system knowledge and estimation have been the targets for successful interventions that have yielded transferable gains in mathematical skills (Griffin, 2005; Griffin, Case, & Siegler, 1994; Opfer & Thompson, 2008; Ramani & Siegler, 2008; Siegler & Ramani, 2008, 2009). No study has reported that increasing the speed of number comparison results in improved mathematical skills. A study that compared the efficacy of interventions targeting different factors could make an important contribution to understanding variation in arithmetic skills and to discriminating between causes and consequences of poor number skills.

## Figures and Tables

**Table 1 tbl1:** Descriptives for Domain-General and Numerical Factors and Arithmetic Skills

Factor and measure	*M*	*SD*	Reliability	Factor ICC
Domain-general				
Working memory: Phonological loop				
Word List Recall (WMTB-C)	19.51	3.41	.82	.17
Working memory: Visuo-spatial sketchpad				.01
Block Recall (WMTB-C)	23.06	4.21	.85	
Mazes Memory (WMTB-C)	12.59	6.65	.92	
Working memory: Central executive				
Listening Recall (WMTB-C)	10.34	3.32	.82	.09
Reasoning				
CPM raw score	29.41	4.51	.83	.16
CPM standard score	105.39	18.32		
Processing speed				−.03
Symbol Matching (WISC)	17.59	4.50	—	
Pair Cancellation (W-J III)	37.79	9.84	—	
RAN				
Rapid Letter Naming (s) (CTOPP)	40.82	11.24	—	.01
Oral language				.03
Receptive vocabulary (BPVS II)	86.19	13.45	.93	
Receptive grammar (TROG-E)	13.41	3.49	.78	
Numerical factors				
Single digit processing				
Numeral comparison				−.02
Response time (ms)	1,351.40	486.20	.95	
Accuracy (%)	86.80	10.57		
Efficiency	1,458.91	711.34		
Quantity enumeration				−.04
Response time (ms)	31,14.30	1252.36	.82	
Accuracy (%)	94.73	9.13		
Efficiency	3,580.26	1581.47		
Multidigit skills				
Number system knowledge	20.68	6.82	.92	.03
Estimation (PAE)	9.28	4.32	.89	.02
Arithmetic skills				
Basic calculation fluency	16.30	6.50	.92	.02
Written arithmetic	8.46	4.63	.92	.18
Arithmetic word problems (WIAT-II UK Mathematical Reasoning)	38.60	7.64	.91	.03
*Note*. *N* = 258 for all measures. Measure of reliability is split-half reliability for number comparison and quantity enumeration and Cronbach’s alpha for other tests. Em dashes in the Reliability column indicate that neither could be calculated. WMTB-C = Working Memory Test Battery for Children (Pickering & Gathercole, 2001); CPM = Raven’s Colored Progressive Matrices (Raven, 2008); WISC = Wechsler Intelligence Scales for Children (Wechsler, 1992); W-J III = Woodcock-Johnson III (Woodcock et al., 2001); RAN = Rapid Automatized Naming; CTOPP = Comprehensive Test of Phonological Processing (Wagner et al., 1999); BPVS II = British Picture Vocabulary Scale (Dunn et al., 1997); TROG-E = Test for Reception of Grammar (Bishop, 2005); PAE = percent absolute error; WIAT-II UK = Wechsler Individual Achievement Test–Second UK Edition (Wechsler, 2005); ICC = intraclass correlation coefficients.				

**Table 2 tbl2:** Correlations Between Factors and Arithmetic Skills

Factor	Domain-general						Single digit		Multidigit		Arithmetic		
	2	3	4	5	6	7	8	9	10	11	12	13	14
Domain-general													
1. WM phonological loop	.26	.43	.31	.33	.14	.45	.15	.17	.30	.22	.27	.35	.40
2. WM visuo-spatial sketchpad		.36	.44	.47	.19	.31	.26	.29	.37	.47	.34	.45	.44
3. WM central executive			.38	.32	.24	.49	.23	.20	.46	.33	.35	.44	.52
4. Reasoning				.43	.02	.55	.20	.23	.46	.53	.35	.53	.58
5. Processing speed					.34	.42	.44	.39	.52	.45	.53	.43	.54
6. RAN						.03	.36	.28	.27	.15	.32	.21	.21
7. Oral language							.20	.16	.45	.40	.38	.49	.59
Single digit processing													
8. Numeral comparison								.47	.45	.33	.48	.34	.40
9. Quantity enumeration									.38	.35	.32	.23	.30
Multidigit skills													
10. Number system knowledge										.65	.72	.63	.71
11. Estimation											.57	.57	.69
Arithmetic skills													
12. Basic calculation fluency												.64	.72
13. Written arithmetic													.69
14. Arithmetic word problems													
*Note*. *N* = 258. WM = working memory; RAN = Rapid Automatized Naming. *r* > .11, *p* < .05; *r* > .15, *p* < .01.													

**Table 3 tbl3:** Fixed Order Regression Analyses Predicting Basic Calculation Fluency

Model	Factors entered into model	*R*^2^	*R*^2^ change	*p*	Model *F*^a^	(*df*)
A-1	Domain-general	.36	.36	<.001	19.93	(7, 250)
A-2	Single digit processing	.41	.05	<.001	19.06	(9, 248)
A-3	Multidigit skills	.59	.18	<.001	32.00	(11, 246)
B-1	Domain-general	.36	.36	<.001	19.93	(7, 250)
B-2	Multidigit skills	.58	.22	<.001	37.79	(9, 248)
B-3	Single digit processing	.59	.01	.047	32.00	(11, 246)
C-1	Single digit processing	.24	.24	<.001	39.80	(2, 255)
C-2	Multidigit skills	.56	.32	<.001	81.41	(4, 253)
C-3	Domain-general	.59	.03	.034	32.00	(11, 246)
D-1	Multidigit skills	.54	.54	<.001	148.49	(2, 255)
D-2	Single digit processing	.56	.02	.001	81.41	(4, 253)
D-3	Domain-general	.59	.03	.034	32.00	(11, 246)
*Note*. Domain-general factors are working memory components (phonological loop, visuo-spatial sketchpad, and central executive), reasoning, processing speed, RAN, and oral language. Single digit processing factors are numeral comparison and quantity enumeration. Multidigit skills are number system knowledge and estimation. RAN = Rapid Automatized Naming.						
^a^ All model *F* ratios are significant at *p* < .001.						

**Table 4 tbl4:** Fixed Order Regression Analyses Predicting Written Arithmetic

Model	Factors entered into model	*R*^*2*^	*R*^*2*^ change	*p*	Model *F*^a^	(*df*)
A-1	Domain-general	.43	.43	<.001	26.98	(7, 250)
A-2	Single digit processing	.45	.02	.015	22.13	(9, 248)
A-3	Multidigit skills	.53	.08	<.001	25.52	(11, 246)
B-1	Domain-general	.43	.43	<.001	26.98	(7, 250)
B-2	Multidigit skills	.53	.10	<.001	30.87	(9, 248)
B-3	Single digit processing	.53	.00	.297	25.52	(11, 246)
C-1	Single digit processing	.12	.12	<.001	17.56	(2, 255)
C-2	Multidigit skills	.44	.32	<.001	50.59	(4, 253)
C-3	Domain-general	.53	.09	<.001	25.52	(11, 246)
D-1	Multidigit skills	.44	.44	<.001	100.91	(2, 255)
D-2	Single digit processing	.44	.00	.557	50.59	(4, 253)
D-3	Domain-general	.53	.09	<.001	25.52	(11, 246)
*Note*. Domain-general factors are working memory components (phonological loop, visuo-spatial sketchpad, and central executive), reasoning, processing speed, RAN, and oral language. Single digit processing factors are numeral comparison and quantity enumeration. Multidigit skills are number system knowledge and estimation. RAN = Rapid Automatized Naming.						
^a^ All model *F* ratios are significant at *p* < .001.						

**Table 5 tbl5:** Fixed Order Regression Analyses Predicting Arithmetic Word Problems

Model	Factors entered into model	*R*^*2*^	*R*^*2*^ change	*p*	Model *F*^a^	(*df*)
A-1	Domain-general	.55	.55	<.001	44.04	(7, 250)
A-2	Single digit processing	.57	.02	.002	37.15	(9, 248)
A-3	Multidigit skills	.69	.12	<.001	50.76	(11, 246)
B-1	Domain-general	.55	.55	<.001	44.04	(7, 250)
B-2	Multidigit skills	.69	.14	<.001	61.56	(9, 248)
B-3	Single digit processing	.69	.00	.260	50.76	(11, 246)
C-1	Single digit processing	.18	.18	<.001	27.58	(2, 255)
C-2	Multidigit skills	.59	.41	<.001	92.24	(4, 253)
C-3	Domain-general	.69	.10	<.001	50.76	(11, 246)
D-1	Multidigit skills	.59	.59	<.001	183.10	(2, 255)
D-2	Single digit processing	.59	.00	.317	92.24	(4, 253)
D-3	Domain-general	.69	.10	<.001	50.76	(11, 246)
*Note*. Domain-general factors are working memory components (phonological loop, visuo-spatial sketchpad, and central executive), reasoning, processing speed, RAN, and oral language. Single digit processing factors are numeral comparison and quantity enumeration. Multidigit skills are number system knowledge and estimation. RAN = Rapid Automatized Naming.						
^a^ All model *F* ratios are significant at *p* < .001.						

**Table 6 tbl6:** Standard and Factor Scores in Single Point Classifications of MLD, LA, and TA Groups and Persistent Subsets

Variable	Single point						Persistent					
	MLD^a^		LA^b^		TA^c^		MLD^d^		LA^e^		TA^f^	
	*M*	*SD*	*M*	*SD*	*M*	*SD*	*M*	*SD*	*M*	*SD*	*M*	*SD*
Standard scores												
IQ	87.07_a_	17.40	93.93_a_	14.55	110.80_b_	16.12	96.36_a_	10.02	100.71_a_	11.91	112.53_b_	14.45
Mathematics achievement	72.79_a_	10.88	86.10_b_	2.27	110.17_c_	13.44	77.91_a_	3.62	86.14_b_	2.27	111.55_c_	13.35
Domain-general												
Phonological loop	−0.88_a_	0.90	−0.33_b_	0.92	0.22_c_	0.92	−0.73_a_	0.95	−0.35_ab_	1.18	0.27_b_	0.93
Visuo-spatial sketchpad	−0.72_a_	1.05	−0.46_a_	0.83	0.22_b_	0.91	−0.51_a_	0.91	−0.30_a_	0.56	0.28_a_	0.91
Central executive	−1.12_a_	0.82	−0.40_b_	0.89	0.26_c_	0.90	−1.09_a_	0.78	−0.55_a_	1.00	0.35_b_	0.88
Reasoning	−0.99_a_	0.87	−0.62_a_	0.80	0.29_b_	0.89	−0.53_a_	0.45	−0.25_a_	0.56	0.40_b_	0.80
Processing speed	−1.07_a_	0.92	−0.49_b_	0.87	0.28_c_	0.89	−0.96_a_	0.70	−0.50_a_	0.96	0.36_b_	0.85
RAN	−0.78_a_	1.00	−0.01_b_	1.11	0.12_b_	0.91	−0.93_a_	0.87	−0.23_ab_	1.32	0.11_b_	0.88
Oral language	−0.98_a_	0.85	−0.49_b_	0.79	0.26_c_	0.93	−0.62_a_	0.60	−0.28_ab_	0.76	0.35_b_	0.90
Single number processing												
Numeral comparison	−1.02_a_	0.91	−0.17_b_	0.97	0.20_b_	0.92	−1.23_a_	0.84	0.00_b_	1.03	0.24_b_	0.93
Quantity enumeration	−0.72_a_	1.18	−0.26_b_	1.00	0.17_b_	0.91	−0.74_a_	1.13	−0.08_ab_	1.18	0.22_b_	0.88
Multidigit												
Number system knowledge	−1.30_a_	0.48	−0.72_b_	0.52	0.36_c_	0.88	−1.47_a_	0.31	−0.82_b_	0.63	0.48_c_	0.83
Estimation	−1.16_a_	0.67	−0.70_b_	0.75	0.34_c_	0.87	−1.27_a_	0.42	−0.34_b_	0.74	0.44_c_	0.84
*Note*. Within a classification, the means in the same row that do not share a subscript differ significantly at *p* < .05 (Ryan-Einot-Gabriel-Welsch or Games-Howell post hoc comparisons). MLD = mathematical learning disability group; LA = low achieving group; TA = typical achieving group; RAN = rapid automatized naming.												
^a^ *n =* 29. ^b^ *n =* 42. ^c^ *n =* 187. ^d^ *n =* 11. ^e^ *n =* 14. ^f^ *n =* 166.
